# Supplementary material for: Alterations in Tumor Aggression Following Androgen Receptor Signaling Restoration in Canine Prostate Cancer Cell Lines
Source: Int J Mol Sci. 2024 Aug 7;25(16):8628. doi: 10.3390/ijms25168628 (PMC11354774; doi:10.3390/ijms25168628)
Supplement: Supplementary file 1 [file ijms-25-08628-s001.zip › ijms-3089421-supplementary.pdf]

```
A
Canine   1 MEVQLGLGRVYPRPPSKTYRGAFQNLFQSVREVIQNPGPRHPEAVSAAPP  50
           ||||||||||||||||||||||||||||||||||||||||||||.|||||
Human    1 MEVQLGLGRVYPRPPSKTYRGAFQNLFQSVREVIQNPGPRHPEAASAAPP  50

B
Canine 433 SSSWHTLFTAEEGQLYGPCGGSGGGSAGDG--------------GSVAPY 468
           |||||||||||||||||||||.|||..|.|              |:||||
Human  432 SSSWHTLFTAEEGQLYGPCGGGGGGGGGGGGGGGGGGGGGGGEAGAVAPY 481

C
Canine 519 SGPYGDMRLETARDHVLPIDYYFPPQKTCLICGDEASGCHYGALTCGSCK 568
           ||||||||||||||||||||||||||||||||||||||||||||||||||
Human  532 SGPYGDMRLETARDHVLPIDYYFPPQKTCLICGDEASGCHYGALTCGSCK 581

Canine 569 VFFKRAAEGKQKYLCASRNDCTIDKFRRKNCPSCRLRKCYEAGMTLGARK 618
           ||||||||||||||||||||||||||||||||||||||||||||||||||
Human  582 VFFKRAAEGKQKYLCASRNDCTIDKFRRKNCPSCRLRKCYEAGMTLGARK 631

D
Canine 569 VFFKRAAEGKQKYLCASRNDCTIDKFRRKNCPSCRLRKCYEAGMTLGARK 618
           ||||||||||||||||||||||||||||||||||||||||||||||||||
Human  582 VFFKRAAEGKQKYLCASRNDCTIDKFRRKNCPSCRLRKCYEAGMTLGARK 631

Canine 619 LKKLGNLKLQEEGEASNVTSPTEEPTQKLTVSHIEGYECQPIFLNVLEAI 668
           ||||||||||||||||:.||||||.|||||||||||||||||||||||||
Human  632 LKKLGNLKLQEEGEASSTTSPTEETTQKLTVSHIEGYECQPIFLNVLEAI 681

E
Canine 819 RMNYIKELDRIIACKRKNPTSCSRRFYQLTKLLDSVQPIARELHQFTFDL 868
           ||||||||||||||||||||||||||||||||||||||||||||||||||
Human  832 RMNYIKELDRIIACKRKNPTSCSRRFYQLTKLLDSVQPIARELHQFTFDL 881
```

Supplementary Figure S1. AR gene homology between canines and humans. A, B) Sequences “FQNLF” and “WHTLF” are NTD sequences that interact with the C-terminal domain after ligand binding and are conserved between the species. **C)** DNA binding domain of AR to AREs in the genome is conserved between the species. **D)** The NLS of AR is also conserved. **E)** Co-activator sequences in the C-terminal domain follow a “LxxLL” motif, and there is evidence of conserved co-activator elements between the species.

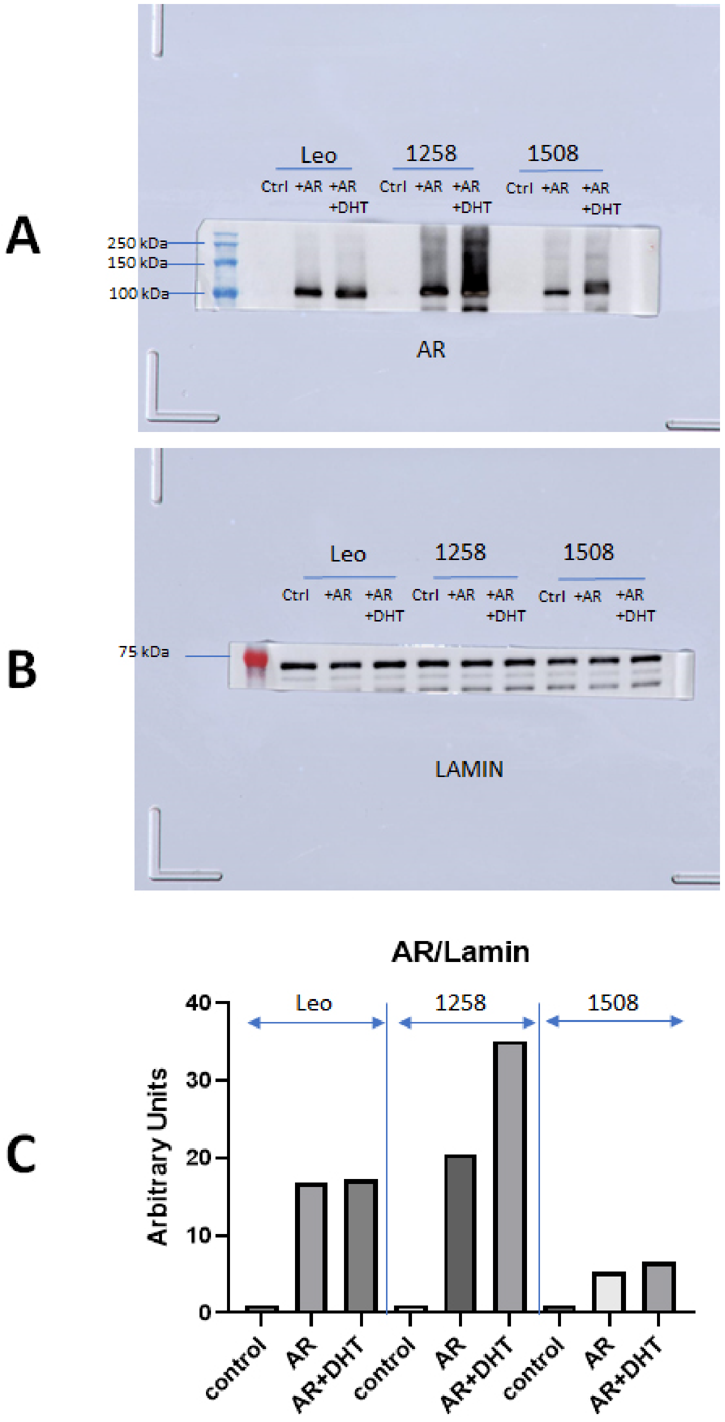

Supplementary Figure S2. Original, uncropped and unadjusted images supporting the Western blot results reported in Figure 2A of the main article. Gel was run and transferred onto a membrane that was cut in order to show AR and loading control lamin on the same gel. Markers are shown. (A) AR blot from Figure 2A, (B) Loading control (C) Quantitation of bands from (A) and (B). Bands were quantitated by Image J, then the raw Lamin numbers for AR and AR+DHT for each cell line was normalized to the corresponding control cells. Then AR levels were normalized to the normalized lamin bands. These results were repeated three times with similar results.
